# Morpholino Gene Knockdown in Adult *Fundulus heteroclitus*: Role of SGK1 in Seawater Acclimation

**DOI:** 10.1371/journal.pone.0029462

**Published:** 2011-12-22

**Authors:** Emily G. Notch, Joseph R. Shaw, Bonita A. Coutermarsh, Marisa Dzioba, Bruce A. Stanton

**Affiliations:** 1 Department of Microbiology and Immunology and of Physiology, Dartmouth Medical School, Hanover, New Hampshire, United States of America; 2 Mount Desert Island Biological Laboratory, Salisbury Cove, Maine, United States of America; 3 The School of Public and Environmental Affairs and The Center for Genomics and Bioinformatics, Indiana University, Bloomington, Indiana, United States of America; Ecole Normale Supérieure de Lyon, France

## Abstract

The Atlantic killifish (*Fundulus heteroclitus*) is an environmental sentinel organism used extensively for studies on environmental toxicants and salt (NaCl) homeostasis. Previous research in our laboratory has shown that rapid acclimation of killifish to seawater is mediated by trafficking of CFTR chloride channels from intracellular vesicles to the plasma membrane in the opercular membrane within the first hour in seawater, which enhances chloride secretion into seawater, thereby contributing to salt homeostasis. Acute transition to seawater is also marked by an increase in both mRNA and protein levels of serum glucocorticoid kinase 1 (SGK1) within 15 minutes of transfer. Although the rise in SGK1 in gill and its functional analog, the opercular membrane, after seawater transfer precedes the increase in membrane CFTR, a direct role of SGK1 in elevating membrane CFTR has not been established *in vivo*. To test the hypothesis that SGK1 mediates the increase in plasma membrane CFTR we designed two functionally different vivo-morpholinos to knock down SGK1 in gill, and developed and validated a vivo-morpholino knock down technique for adult killifish. Injection (intraperitoneal, IP) of the splice blocking SGK1 vivo-morpholino reduced SGK1 mRNA in the gill after transition from fresh to seawater by 66%. The IP injection of the translational blocking and splice blocking vivo-morpholinos reduced gill SGK1 protein abundance in fish transferred from fresh to seawater by 64% and 53%, respectively. Moreover, knock down of SGK1 completely eliminated the seawater induced rise in plasma membrane CFTR, demonstrating that the increase in SGK1 protein is required for the trafficking of CFTR from intracellular vesicles in mitochondrion rich cells to the plasma membrane in the gill during acclimation to seawater. This is the first report of the use of vivo-morpholinos in adult killifish and demonstrates that vivo-morpholinos are a valuable genetic tool for this environmentally relevant model organism.

## Introduction

The Atlantic killifish (*Fundulus heteroclitus*) is an environmental sentinel organism used extensively for studies of environmental toxicants and salt (NaCl) homeostasis [1]–[7]. Killifish are euryhaline teleosts that live primarily in estuaries where they are exposed to rapid changes in salinity. Acclimation to increased salinity involves the remodeling of mitochondrion rich cells in the opercular membrane and gill from ion absorption to ion (NaCl) secretion in order to maintain osmotic balance [8]. In the seawater gill chloride (Cl^−^) secretion is a two-step process; uptake across the basolateral membrane via the Na^+^, K,^+^ 2Cl^−^ co-transporter, followed by secretion of Cl^−^ across the apical membrane through the cystic fibrosis transmembrane conductance regulator (CFTR) [9], [10]. Na^+^, K^+^-ATPase is also necessary to maintain a low intracellular sodium concentration, which facilitates Cl^−^ uptake through the Na^+^, K^+^, 2Cl^−^ co-transporter [6]. Sodium is thought to be secreted passively across the paracellular pathway, driven by the negative transepithelial potential established by Cl^−^ secretion. CFTR is a cAMP regulated Cl^−^ channel that belongs to the ATP-binding cassette family of proteins, whose mutations result in cystic fibrosis the most common lethal autosomal recessive disorder in Caucasians [11].

Acclimation to seawater in killifish occurs in two distinct stages. In the long term (>8 hours in seawater), an increase in plasma cortisol activates the glucocorticoid receptor, which increases CFTR mRNA and protein levels in the opercular membrane and gill [4]. In the short term (15 minutes to 1 hour in seawater), recent studies by our laboratory have demonstrated an increase in CFTR mediated Cl^−^ secretion by the opercular epithelium, which is functionally equivalent to the gill [5]. The rise in Cl^−^ secretion occurs in concert with an increase in plasma membrane CFTR, with no change in total CFTR abundance, revealing that the short term increase in Cl^−^ secretion is mediated, at least in part, by trafficking of CFTR from intracellular vesicles to the plasma membrane [5].

Several lines of evidence suggest that serum glucocorticoid kinase 1 (SGK1) may mediate the acute increase in plasma membrane CFTR in killifish gill. First, the increase in membrane CFTR and Cl^−^ secretion in the gill are preceded by a rise in mRNA and protein abundance of SGK1 [5]. Second, SGK1 is a widely expressed, serine/threonine kinase that is transcriptionally activated by a variety of environmental stressors including plasma hypertonicity, hormones, and cAMP [12], [13]. Third, SGK1 is regulated at both the translational and post-translational levels, with mRNA induction and downstream phosphorylation of target proteins within minutes of plasma hypertonicity [12]–[14]. Fourth, SGK1 increases plasma membrane abundance of other ion channels, including the epithelial Na^+^ channel (ENaC) [14]–[16]. Finally, killifish SGK1 induces an equivalent increase in CFTR Cl^−^ currents and CFTR abundance in the plasma membrane of *Xenopus* oocytes [17], [18]. Collectively these observations support, but do not prove, the hypothesis that short term acclimation to seawater involves a rapid increase in SGK1, which mediates the trafficking of CFTR from intracellular vesicles to the plasma membrane, resulting in enhanced CFTR mediated Cl^−^ secretion across killifish opercular membrane and gill. To test this hypothesis directly we developed a gene knock down approach using anti-sense vivo-morpholino technology in adult killifish. Morpholinos are anti-sense oligonucleotide analogs that bind to complementary RNA sequences and inhibit processing of mRNA, either translation or splicing of pre-mRNA, by steric hindrance [19]. Since standard morpholino oligonucleotides do not cross plasma membranes efficiently, this study utilized vivo-morpholinos (Gene Tools Inc), which are morpholino oligonucleotides coupled to eight guanidinium head groups that facilitate cellular uptake by endocytosis [19], [20]. Application of morpholino technology to knock down specific targets has been used in zebrafish and killifish embryos [21], adult zebrafish [22], [23], and *Xenopus* oocytes [19], [24], [25], but not in adult killifish. Although killifish are a valuable environmental model and are used extensively to study acclimation to seawater (>800 publications in a recent PubMed search), there is limited information on the killifish transcriptome or genome and few genetic tools available for killifish that make other fish models, such as zebrafish and medaka, more easily manipulated.

The goal of this study was two-fold; to develop a method using IP injection of vivo-morpholinos to selectively knock down target genes in adult killifish, and to use this method to test the hypothesis that SGK1 mediates the rapid (1 hour) increase in plasma membrane CFTR in the gill when killifish are transferred from freshwater to seawater. To these ends we designed two functionally different vivo-morpholinos to knock down SGK1, and developed and validated a vivo-morpholino knock down technique for adult killifish. Injection (IP) of either a translational blocking or a splice blocking vivo-morpholino blocked the increase in SGK1 protein abundance in fish transferred from freshwater to seawater and completely eliminated the seawater induced rise in plasma membrane CFTR in gill, demonstrating that the increase in SGK1 protein is required for the trafficking of CFTR from intracellular vesicles in mitochondrion rich cells to the plasma membrane during acclimation to seawater. The development of the use of vivo-morpholinos in adult killifish provides a novel and valuable genetic tool for this environmentally relevant model organism.

## Results

### Vivo-morpholino knock down of SGK1

**Figure 1:**
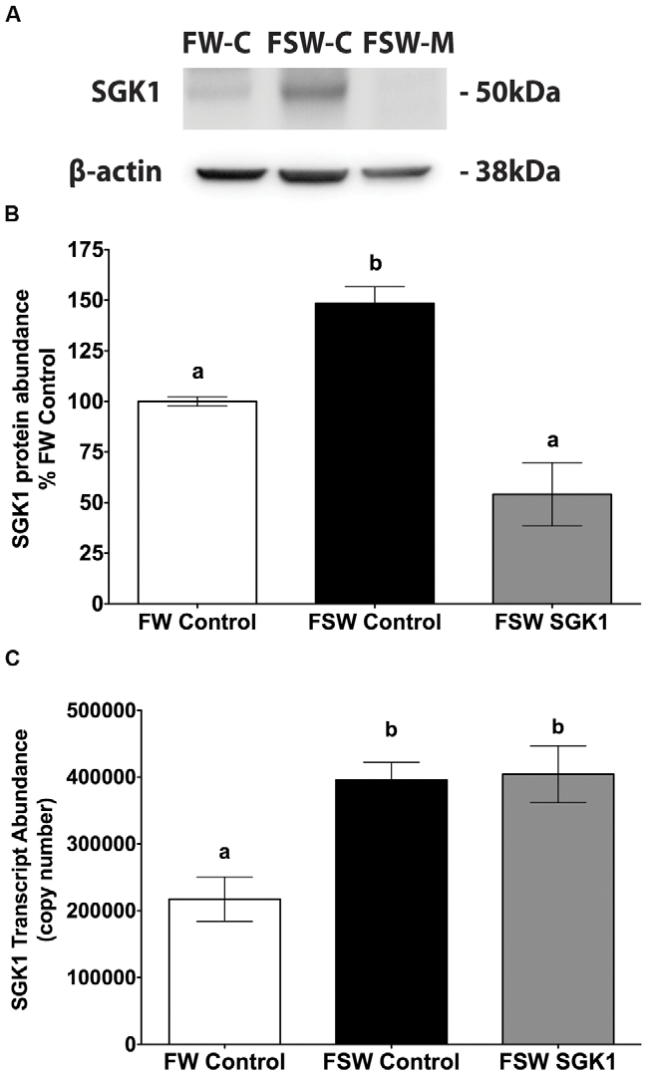
SGK1 protein and mRNA levels in gill of *Fundulus heteroclitus* injected with the SGK1 translation blocking vivo-morpholino. **A**: Representative Western blot of SGK1. **B**: Summary of SGK1 Western blot experiments. **C**: SGK1 mRNA abundance. Freshwater acclimated fish were injected with 14 µg/g SGK1 vivo-morpholino, or control vivo-morpholino. Four hours after injection fish were transferred to seawater for 1 h. n = 5. Different letters indicate statistically significant treatment means p<0.05. FW-Control: Freshwater control vivo-morpholino, FSW-Control: Freshwater to seawater control vivo-morpholino, FSW SGK1: Freshwater to seawater SGK1 vivo-morpholino.

Freshwater acclimated adult killifish were intraperitoneal (IP) injected with 14 µg/g SGK1 translational blocking vivo-morpholino, or 14 µg/g of control vivo-morpholino, and returned to freshwater for 4 hours. Fish were then transferred to seawater for 1 h, the time when SGK1 protein increases to its maximum level [5]. Freshwater control vivo-morpholino treated fish remained in freshwater for a total of 5 hours. The SGK1 translational blocking vivo-morpholino elicited a significant, 64% reduction (p<0.05) in gill SGK1 protein in fish transferred from freshwater to seawater compared to SGK1 in adult killifish injected with the same concentration of control vivo-morpholino, which had a 1.5 fold increase (p<0.05) in SGK1 protein, a result comparable to a previous study (Figure 1A and 1B) [5]. As expected, the translational blocking vivo-morpholino, which inhibits protein synthesis by steric hindrance of the ribosomal complex, had no effect on SGK1 mRNA levels. Compared to fish injected with the control vivo-morpholino and maintained in freshwater, SGK1 mRNA significantly increased 1.8 fold (p<0.05) in killifish injected with either control vivo-morpholino or the SGK1 vivo-morpholino and then transferred from freshwater to seawater for 1 h (Figure 1C). Due to low basal levels of SGK1 protein in freshwater acclimated killifish, no difference was observed in SGK1 gill protein levels between freshwater control vivo-morpholino and freshwater SGK1 vivo-morpholino (108% of control).

**Figure 2:**
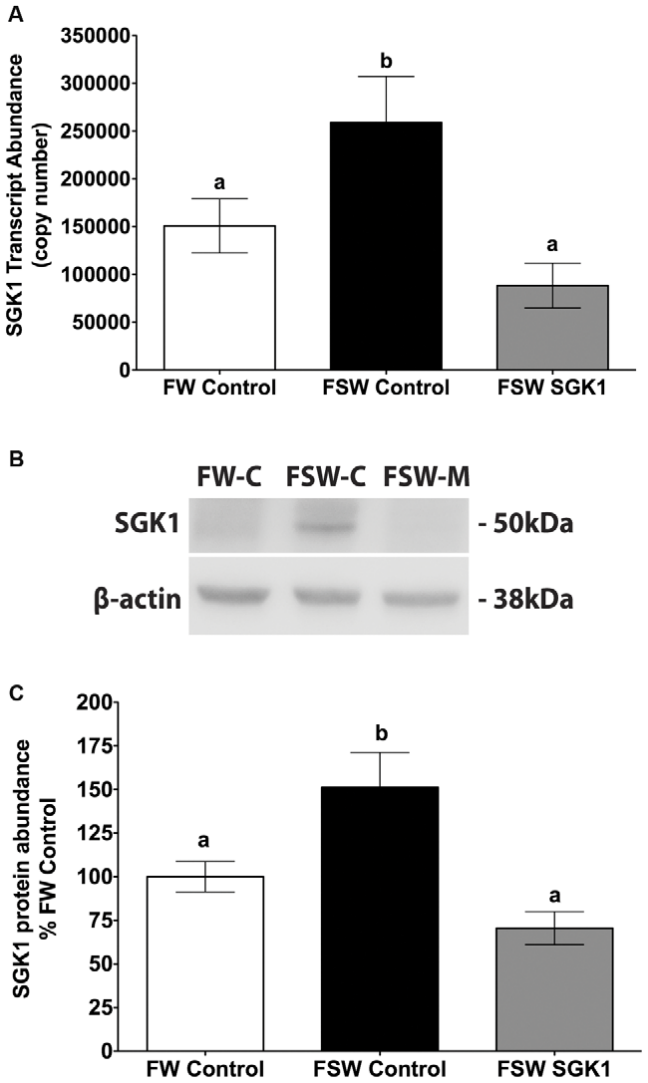
SGK1 mRNA and protein levels in gills of adult *Fundulus heteroclitus* injected with splice blocking SGK1 vivo-morpholino. **A**: SGK1 mRNA abundance **B**: Representative Western blot of SGK1. **C**: Summary of SGK1 Western blot experiments. Freshwater acclimated fish were injected with 20 µg/g SGK1 vivo-morpholino, control vivo-morpholino or PBS control. Four hours after injection fish were transferred to seawater for 1 h. n = 5. Different letters indicate statistically significant treatment means p<0.05. FW-Control: Freshwater control vivo-morpholino, FSW-Control: Freshwater to seawater control vivo-morpholino, FSW SGK1: Freshwater to seawater SGK1 vivo-morpholino.

Studies were also done using a splice blocking SGK1 vivo-morpholino. Since information on the killifish genome is limited, SGK1 exon-intron junctions were predicted and primers designed based on alignment with conserved regions of zebrafish and medaka genomic DNA sequence. Primers listed in Table 1 were used to amplify killifish genomic DNA across predicted exon-intron junctions to obtain sequence information for vivo-morpholino design. Based on the killifish intron sequence, a splice blocking SGK1 vivo-morpholino was developed to excise exon 3 by steric hindrance of small nuclear ribonucleoproteins at the 3′ acceptor sequence. Subsequently, freshwater acclimated adult killifish were IP injected with the 20 µg/g SGK1 splice blocking vivo-morpholino, or 20 µg/g of the control vivo-morpholino, and returned to freshwater for 4 hours. Fish were then transferred to seawater for 1 h, the time when SGK1 protein achieves the maximum level [5]. Freshwater control vivo-morpholino treated fish remained in freshwater for a total of 5 hours. The splice blocking SGK1 vivo-morpholino elicited a significant, 66% decrease (p<0.05) in gill SGK1 mRNA in seawater fish compared to fish injected with control vivo-morpholino and transferred to seawater (Figure 2A). Moreover, the splice blocking SGK1 vivo-morpholino significantly reduced the increase of SGK1 protein levels in the gill in comparison to fish injected with control vivo-morpholino (53% reduction, p<0.05, Figure 2B and 2C).

**Table 1:** Primer sets used for amplification of Killifish SGK1 intron and exon boundaries and for qPCR.

Exon	Sequence	Purpose
6 Fwd	5′ GGCCTGCATTATTCTTTCCA	qPCR
6 Rev	5′GCAGGATCTTCTCTGGCTTC	qPCR
2 Fwd	5′ AACAAAGGAGGATGGGTCTG	qPCR/intron-exon boundaries
3 Rev	5′GGAGGAGAGGGGTTTGAGTT	qPCR/intron-exon boundaries

Because both SGK1 vivo-morpholinos were equally effective at preventing the induction of SGK1 in the gill of adult fish transitioned from freshwater to seawater for 1 hour, the remaining studies were conducted using the translational blocking SGK1 vivo-morpholino.

### SGK1 knock down in liver and intestine

**Figure 3:**
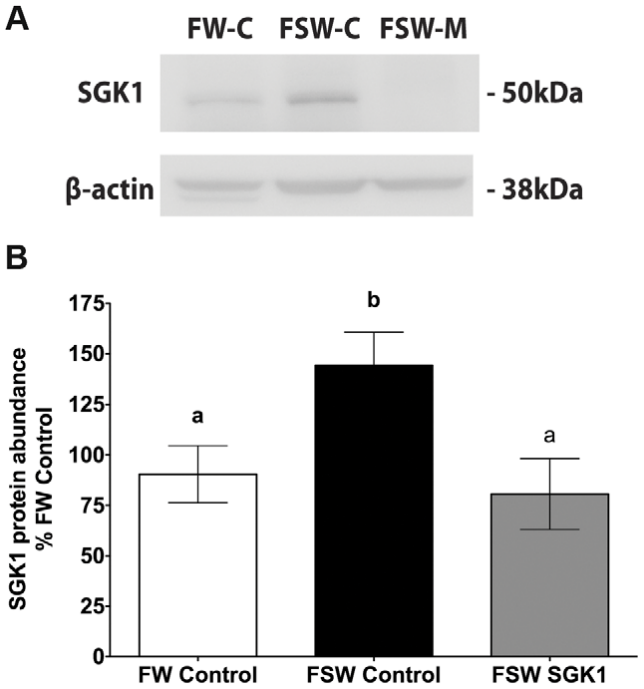
SGK1 protein levels in intestine of *Fundulus heteroclitus* injected with the translational blocking vivo-morpholino. **A**: Representative Western blot of SGK1 in intestine. **B**: Summary of SGK1 Western blot experiments in intestine. Freshwater acclimated fish were injected with 14 µg/g SGK1 vivo-morpholino, or control vivo-morpholino. Eight hours after injection fish were transferred to seawater for 1 h. n = 5. Different letters indicate statistically significant treatment means p<0.05. FW-Control: Freshwater control vivo-morpholino, FSW-Control: Freshwater to seawater control vivo-morpholino, FSW SGK1: Freshwater to seawater SGK1 vivo-morpholino.

**Figure 4:**
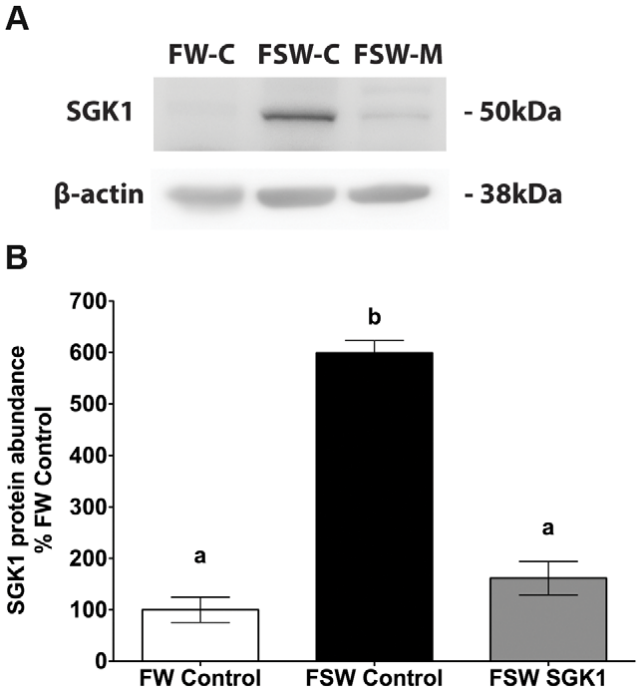
SGK1 protein levels in liver of *Fundulus heteroclitus* injected with the translational blocking vivo-morpholino. **A**: Representative Western blot of SGK1 in liver. **B**: Summary of SGK1 Western blot experiments in liver. Freshwater acclimated fish were injected with 14 µg/g SGK1 vivo-morpholino, or control vivo-morpholino. Eight hours after injection fish were transferred to seawater for 1 h. n = 5. Different letters indicate statistically significant treatment means p<0.05. FW-Control: Freshwater control vivo-morpholino, FSW-Control: Freshwater to seawater control vivo-morpholino, FSW SGK1: Freshwater to seawater SGK1 vivo-morpholino.

Studies were also conducted to determine if the SGK1 translation blocking vivo-morpholino inhibited the increase in SGK1 in liver and intestine when fish were transferred from fresh water to seawater. The SGK1 translational blocking vivo-morpholino significantly reduced the increase in SGK1 protein levels in liver and intestine when fish were transferred from fresh water to seawater (p<0.05, Figure 3A–B and 4A–B).

### Duration of SGK1 knock down in gills

**Figure 5:**
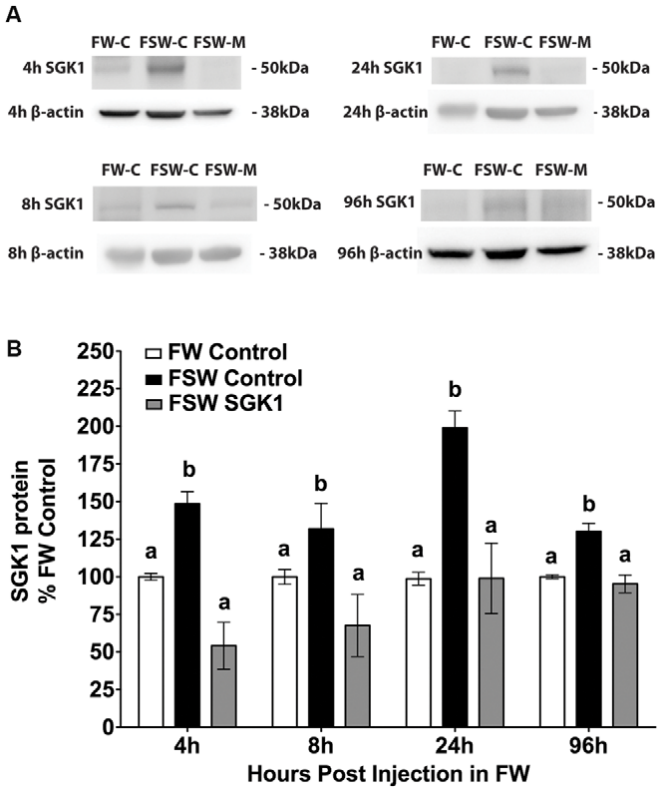
Time course of SGK1 protein knock down in the gill of fish injected with the translational blocking vivo-morpholino. Fish were injected one time with 14 µg/g SGK1 vivo-morpholino, control vivo-morpholino or PBS and placed in freshwater for 4, 8, 24, or 96 h and then transferred to seawater for 1 h. **A**: Representative Western blots **B**: Summary of SGK1 protein levels. n = 5 per time point. Different letters indicate statistically significant differences between treatment means for a given time point. p<0.05. FW-C: Freshwater control vivo-morpholino, FSW-C: Freshwater to seawater control vivo-morpholino, FSW-M: Freshwater to seawater SGK1 vivo-morpholino.

The next series of studies were designed to determine the duration of SGK1 knock down in the gills achieved by IP injection of vivo-morpholinos. Fish were IP injected with 14 µg/g of the translational blocking SGK1 or 14 µg/g of the control vivo-morpholino and then returned to freshwater for 4 to 96 h post injection. After 4, 8, 24 or 96 h in freshwater, vivo-morpholino injected fish were transitioned to seawater for 1 h. Compared to control vivo-morpholino, SGK1 vivo-morpholino decreased SGK1 protein at all time points studied in fish transferred to seawater (p<0.05) (Figure 5). At all time points tested, the SGK1 vivo-morpholino completely blocked the seawater induced increase in SGK1 protein abundance in the gill.

### Plasma membrane CFTR and Na^+^, K^+^-ATPase: Role of SGK1

**Figure 6:**
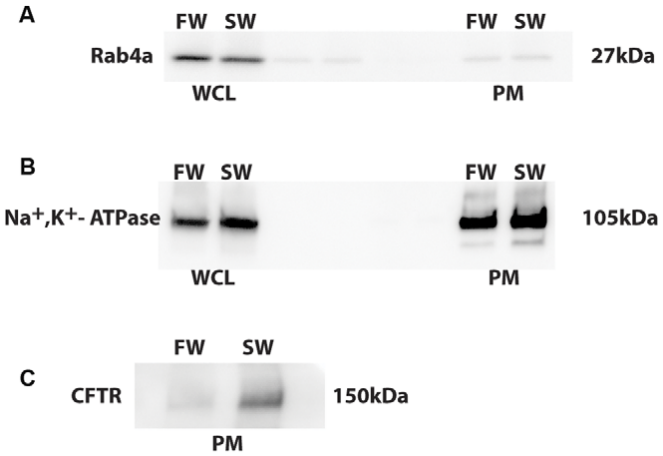
Validation of plasma membrane preparation. Representative Western blots of whole cell lysates (WCL) and plasma membrane preparations (PM) from freshwater or seawater acclimated killifish for: **A**: Rab4a. **B**: Na^+^, K^+^-ATPase and **C**: CFTR. FW: freshwater, SW: seawater acclimated fish. Equal volumes of WCL and PM were loaded in each lane.

To test the hypothesis that SGK1 mediates the rapid (1 hour) increase in plasma membrane CFTR when killifish are transferred from freshwater to seawater, plasma membranes were isolated from killifish gill using a protocol adapted from Flik et. al. [26], as described in Methods, and Western blot studies were conducted to measure CFTR in plasma membrane preparations. The protocol for the isolation of plasma membranes was evaluated by examining the presence of Rab4a, which is localized to early endosomes [27], but not in plasma membranes. Compared to the entire cell lysate, which contains both plasma membranes and cytosolic proteins like Rab4a, isolated plasma membranes from gill contained 85% less Rab4a in both freshwater and saltwater acclimated fish, a value consistent with that reported by Flik et al (Figure 6A) [26], [27]. Moreover, Na^+^, K^+^-ATPase, a plasma membrane protein, was enriched by 1.5 and 1.4 fold in isolated plasma membranes compared to entire cell lysates in freshwater and saltwater fish, respectively (Figure 6B). Taken together, these results demonstrate that the protocol adapted from Flick et al [26] significantly enriched plasma membranes with minimal contamination of cytosolic proteins.

**Figure 7:**
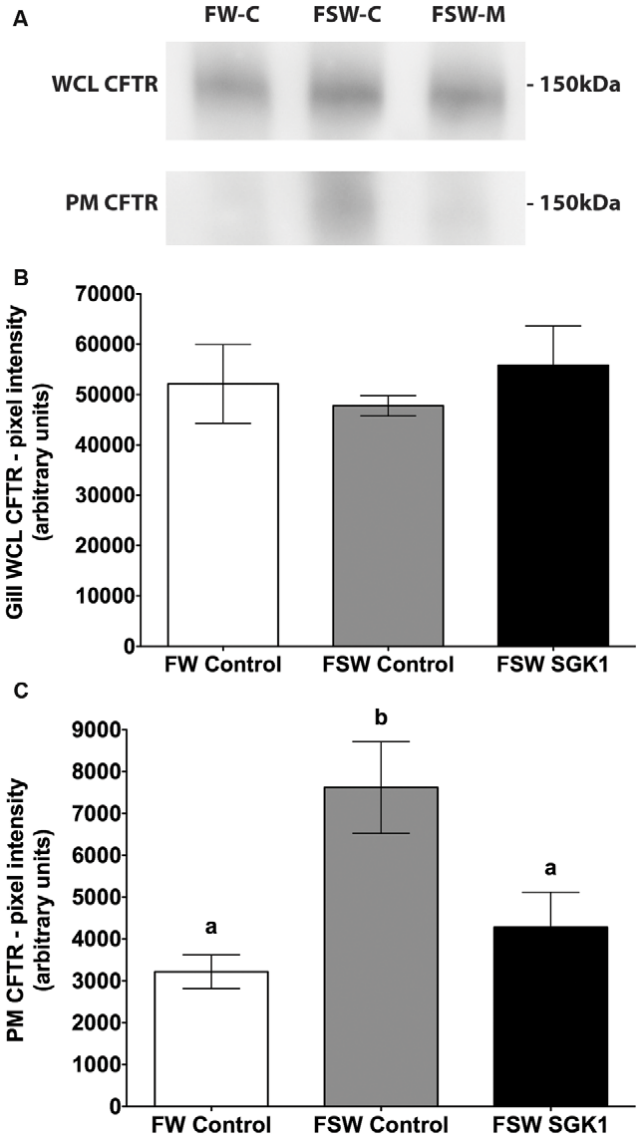
CFTR protein levels in gill of fish injected with SGK1 vivo-morpholino. Freshwater acclimated fish were injected with 14 µg/g SGK1 or control vivo-morpholino. Four hours after injection fish were transferred to seawater for 1 h. The increase in plasma membrane CFTR in seawater fish compared to freshwater fish was not due to differences in contamination of the membrane preparation by cytosolic proteins (i.e., CFTR), since the amount of Rab4a in each preparation was minimal, and equivalent in all membrane preparations (see Figure 4A). **A**: Representative Western blot of CFTR. **B**: Summary of CFTR WCL protein levels. **C**: Summary of CFTR PM protein levels. n = 4. Different letters indicate statistically significant treatment means p<0.05. FW-C: Freshwater control vivo-morpholino, FSW-C: Freshwater to seawater control vivo-morpholino, FSW-M: Freshwater to seawater SGK1 vivo-morpholino, WCL: whole cell lysate, PM: plasma membrane.

**Figure 8:**
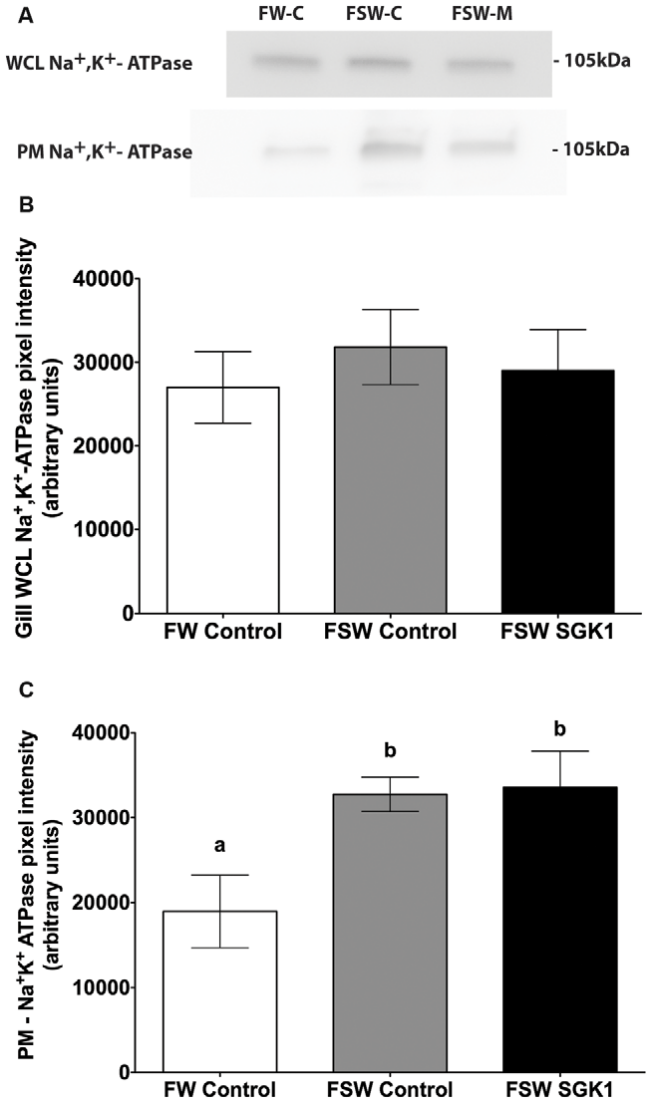
Na^+^, K^+^-ATPase protein levels in gill of fish injected with SGK1 vivo-morpholino. Freshwater acclimated fish were injected with 14 µg/g SGK1 or control vivo-morpholino. Four hours after injection fish were transferred to seawater for 1 h. The increase in plasma membrane Na^+^, K^+^-ATPase in seawater fish compared to freshwater fish was not due to differences in contamination of the membrane preparation by cytosolic proteins, since the amount of Rab4a in each preparation was minimal, and equivalent in all membrane preparations (see Figure 4A). **A**: Representative Western blot of Na^+^, K^+^-ATPase. **B**: Summary of Na^+^, K^+^-ATPase WCL protein levels. **C**: Summary of Na^+^, K^+^-ATPase PM protein levels. n = 4. Different letters indicate statistically significant treatment means p<0.05. FW-C: Freshwater control vivo-morpholino, FSW-C: Freshwater to seawater control vivo-morpholino, FSW-M: Freshwater to seawater SGK1 vivo-morpholino, WCL: whole cell lysate, PM: plasma membrane.

Western blot studies were subsequently performed on plasma membrane preparations to determine first if transfer from freshwater to seawater increased plasma membrane CFTR and, second, if the SGK1 vivo-morpholino blocked the increase in plasma membrane CFTR when fish were transferred from freshwater to seawater. Plasma membrane CFTR in gill significantly increased in control vivo-morpholino fish transferred from freshwater to seawater, without a change in total CFTR abundance (p<0.05) (Figure 7A–C). The SGK1 vivo-morpholino reduced plasma membrane CFTR by 44% (p<0.05) in seawater fish compared the seawater fish injected with the control vivo-morpholino (Figure 7A and 7C). However, the translation blocking SGK1 vivo-morpholino had no effect on Na^+^, K^+^-ATPase when fish were transferred from freshwater to seawater. Plasma membrane Na^+^, K^+^-ATPase increased significantly and to the same extent in both control vivo-morpholino and SGK1 vivo-morpholino injected fish transferred from freshwater to seawater in comparison to freshwater control vivo-morpholino fish, without a change in whole cell Na^+^, K^+^-ATPase abundance (p<0.05) (Figure 8A–C).

## Discussion

The major contributions of this paper are three-fold. First it provides direct evidence that SGK1 is involved in the rapid (1 hour) trafficking of CFTR from intracellular vesicles to the plasma membrane in gill mitochondrion rich cells of killifish during acclimation to seawater. Second it describes a novel genetic tool to block gene expression in adult killifish using vivo-morpholino antisense oligonucleotides. Finally, the research presented here describes a modified technique to measure plasma membrane proteins, including CFTR, in killifish gill, eliminating the need to conduct studies in surrogate tissues such as the opercular membrane.

The role of SGK1 in mediating the movement of CFTR from intracellular vesicles to the plasma membrane is consistent with the hypothesis set forth for these studies and based on a previous report in *Xenopus* oocytes [17]. In heterologous expression studies using *Xenopus* oocytes, killifish SGK1 increased plasma membrane CFTR abundance and CFTR Cl^−^ currents [17]. However, the present study provides the first direct evidence *in vivo* demonstrating that the rise in SGK1 immediately following transition to seawater is necessary for trafficking of CFTR from an intracellular pool to the plasma membrane in the gill of adult killifish.

Studies were also conducted to examine the effect of knock down of SGK1 on plasma membrane Na^+^, K^+^-ATPase. The rational for this experiment was three-fold: (1) SGK1 increases plasma membrane Na^+^, K^+^-ATPase abundance in *Xenopus* oocytes [28], (2) SGK1 increases when fish are transferred from freshwater to seawater, and (3) freshwater to seawater transfer increases the enzyme activity of Na^+^, K^+^-ATPase in killifish gill [6]. Our studies, presented in Figures 8A–C, demonstrate that freshwater to seawater transfer increased plasma membrane Na^+^, K^+^-ATPase, but that the SGK1 vivo-morpholino did not block the increase in plasma membrane Na^+^, K^+^-ATPase. Taken together, this experiment demonstrates that the SGK1 vivo-morpholino did not have an off target effect on Na^+^, K^+^-ATPase, and that increased SGK1 does not mediate the rise in plasma membrane Na^+^, K^+^-ATPase when fish are transferred from freshwater to seawater. This is the first report of rapid (1 hour) trafficking of Na^+^, K^+^-ATPase to the plasma membrane in the killifish gill when fish are transferred from freshwater to seawater, which provides an important insight into the mechanism of acclimation of mitochondrion rich cells to increased salinity.

This study reports the first use of vivo-morpholinos for targeted knock down of gene expression in adult killifish, an environmental sentinel organism. We designed two functionally different vivo-morpholinos to block the increase in SGK1 observed in fish transferred to seawater, and developed and validated a vivo-morpholino knock down technique for adult killifish. Injection (IP) of adult killifish with the splice blocking SGK1 vivo-morpholino resulted in a significant, 66% reduction in SGK1 mRNA in the gill after transition from freshwater to seawater. The SGK1 vivo-morpholinos also reduced SGK1 protein levels in the gill by 64% (translational blocking) and 53% (splice blocking). Importantly, knock down of SGK1 prevented the increase in plasma membrane CFTR in gills when fish were transferred to seawater, confirming our hypothesis that the increase in SGK1 protein is required for the trafficking of CFTR from intracellular vesicles in mitochondrion rich cells to the plasma membrane during acclimation to seawater.

This study also describes an adaptation of techniques to measure plasma membrane proteins in killifish gill, which eliminates the need to work in surrogate tissues in killifish such as the opercular membrane, which is functionally analogous to the gill, but is not as quantitatively important as the gill in contributing to salt (NaCl) homeostasis [2], [7], [29], [30]. In a previous study by our laboratory we could not measure plasma membrane proteins in the killifish gill by cell surface biotinylation, most likely due to the thick mucus layer that overlies the gill [5]. Thus, isolation of the plasma membrane of the gill provides a new experimental approach to study membrane proteins in the gill of killifish.

Killifish are commonly used in a variety of toxicological, osmoregulatory, and evolutionary studies due to their widespread distribution in estuaries of differing salinities, contaminant load and environmental toxicants [1], [31]–[34]. While vivo-morpholinos have been recently adapted for use in killifish embryos, this is the first study to use vivo-morpholinos in adult killifish [21]. In fact, very few studies have been published using vivo-morpholinos in adult fish of any species and none using the IP injection technique described in this study [22], [23]. Maki et. al. used IP injection of vivo-morpholinos to knock down Histone B4 in the regenerating lens of newts, showing the ability to use IP injection to knock down targets far from the injection site [35]. In this study IP injection of vivo-morpholino resulted in significant knock down of SGK1 protein in the gill, liver and intestine of killifish. Because the SGK1 vivo-morpholino was able to block the increase in SGK1 in seawater fish even 4 days after injection (Figure 5), our data suggest that the use of vivo-morpholinos are likely to be effective even for genes whose proteins have a very long half-life. The ability to selectively knock down target gene expression with vivo-morpholinos in adult fish provides an extremely valuable genetic tool for killifish, as well as other model teleosts. The knock down of SGK1 also reveals that rapid acclimation to seawater requires an increase in SGK1, which leads to a substantial increase in plasma membrane CFTR in the gill of killifish providing further insight into the mechanisms of acclimation of mitochondrion rich cells to increased salinity.

## Materials and Methods

### Adult killifish

All studies were performed in compliance with Institutional Animal Care and Use Committee guidelines approved by both the Mount Desert Island Biological Laboratory (10-01) and Dartmouth Medical School (10-03-03). *Fundulus heteroclitus* were collected from Northeast Creek (Bar Harbor, ME) and held in aquaria containing running seawater (27ppt) at MDIBL (Salisbury Cove, ME) or artificial seawater at Dartmouth Medical School. Fish were acclimated to freshwater (0.3ppt) by transitioning them to 10% seawater (3ppt) for 2 weeks and then replacement of 10% seawater with “soft” freshwater (48 mg/L NaHCO_3_, 30 mg/L CaSO_4_, 30 mg/L MgSO_4_, 2 mg/L KCl, pH 7.5–8.0) for 2 weeks prior to any experiments [36], [37]. Both male and female fish were used in this study. All fish utilized were sexually mature adults ranging in size from 2–6 grams.

### Vivo-morpholino design

The translational blocking SGK1 vivo-morpholino (5′GAGTTCGAGATCCGCACTCATCAAA) was designed to be complimentary to the translational start of *F. heteroclitus* SGK1 (Accession number AY800243). The splice blocking SGK1 vivo-morpholino (5′CACTTCAGGACTGGAAAAAGAACAA) was designed to target the intron2-exon3 junction. To obtain intron-exon junction sequences, *F. heteroclitus* SGK1 was aligned with Zebrafish (ENSDART00000035021) and Medaka (ENSORLT00000020684) SGK1 to predict exon boundaries. Primers designed to amplify these junctions are listed in Table 1. PCR amplification of cDNA and genomic DNA from killifish gills with corresponding primer sets yielded single, differentially sized bands. Bands from genomic DNA amplifications were excised and sequenced. Alignment of sequence from genomic DNA amplification with known SGK1 sequence provided intron-exon junctions that were submitted to Gene Tools (Philomath, OR) for vivo-morpholino design. For all experiments, standard vivo-morpholino control (5′CCTCCTACCTCAGTTCCAATTTATA) was injected at the same concentration as SGK1 vivo-morpholino.

### Vivo-morpholino injection

Freshwater acclimated adult killifish were intraperitoneal (IP) injected with 14 µg/g SGK1 translational blocking vivo-morpholino, or 20 µg/g SGK1 splice blocking vivo-morpholino or an appropriate concentration of control vivo-morpholino. Fish were returned to freshwater for 4 h, 8 h, 24 h, or 96 h with temperature control (13–15°C) and aeration. After appropriate time in freshwater, fish were transferred to seawater for 1 h, except for freshwater control vivo-morpholino treated fish that remained in freshwater.

### Western blot analysis

Fish were sacrificed by cervical dislocation, and gills, livers and intestine surgically removed and homogenized according to standard protocols in lysis buffer (25 mM Hepes, 10% v/v glycerol, and 1% v/v Triton-X with Complete Protease Inhibitor (Roche Applied Science, Indianapolis, IN) and Halt phosphatase inhibitor (Pierce, Rockford, IL)) for western blotting [4], [5]. Samples were manually homogenized and spun at 14,000×g for 10 min in a refrigerated centrifuge. Protein samples were combined with an equal volume of Laemmli buffer with 1 mM DTT and heat denatured at 85°C for 5 minutes. Samples were stored at −20°C until time of analysis. Western blot analysis of CFTR (Clone ACL-006, 1∶500 dilution, Alomone Labs, Jerusalem, Israel), SGK1 (anti-SGK #S5188, 1∶2000 dilution, Sigma-Aldrich, St. Louis, MO), Na^+^, K^+^-ATPase (Na^+^, K^+^-ATPase a5 supernatant, 1 µg/ml, Developmental Studies Hybridoma Bank, University of Iowa, Iowa City, IA), Rab4a (SC-312, 1∶500 dilution, Santa Cruz Biotechnology, Santa Cruz, CA) and β-actin (Clone C4, 1∶2000 dilution, MP Biomedicals, Solon, OH) in gill lysates was performed as previously described [4], [5]. Previous studies by our laboratory have demonstrated that these antibodies recognize killifish CFTR, Na^+^, K^+^-ATPase, SGK1 and β-actin [4], [5].

### RNA isolation

Fish were sacrificed by cervical dislocation, and gills were excised, rinsed and placed in RNAlater (Ambion, Austin, TX) until time of use. RNA was isolated using the RNeasy kit (Qiagen, Valencia, CA). Briefly, gill samples were thoroughly homogenized with a Tissue-Tearor (Biospec Products, Bartlesville, OK) in lysis buffer containing guanidine-thiocyanate and then mixed with an equal volume of ethanol. Samples were immediately bound to a glass fiber filter and washed three times prior to elution with nuclease free water. Samples were immediately DNAse treated (DNAFree, Ambion, Austin, TX). RNA integrity and concentration was assessed using micro-capillary electrophoresis on an Agilent 2100 Bioanalyzer. RNA was compared to a RNA ladder with 6 RNA transcripts of varying sizes and known concentration of 150 ng/µL. RNA quality was verified by observation of corresponding 18 S and 28 S peaks on the electropherogram. Only intact RNA was used for further analysis.

### qPCR

For quantitative PCR (qPCR) cDNA was synthesized from1 µg of total RNA using Retroscript Reverse Transcriptase (Ambion, Austin, TX) with random decamers. For fluorescence based qPCR reactions Sure start Taq Polymerase (Stratagene, Santa Clara, CA), SYBR green (Stratagene Brilliant SYBR), 300 nM Reference dye, 2.5 mM MgCl_2,_ 100 ng cDNA, 36–60 nM primers and nuclease free water were amplified with initial denaturing at 95°C for 10 min, followed by 40 cycles of 30 sec at 95°C and 1 min at 55°C. To examine SGK1 mRNA levels after injection with the translational blocking vivo-morpholino specific primers (see Table 1) were designed using the known *F. heteroclitus* SGK1 sequence (Accession number AY800243). The forward primer sequence was 5′ GGCCTGCATTATTCTTTCCA and the reverse primer sequence was 5′ GCAGGATGTTCTCTGGCTTC. These primers amplified a 207 bp fragment. To examine SGK1 mRNA levels after injection with the splice blocking vivo-morpholino, primers were designed with the forward primer in exon 2 and the reverse primer in exon 3, using the killifish SGK1 sequence (Accession number AY800243). The forward primer sequences was 5′ AACAAAGGAGGATGGGTCTG and the reverse primer sequence was 5′ GGAGGAGAGGGGTTTGAGTT. These primers amplified a 141 bp fragment. Both sets of primers yielded a single amplicon that was sequenced. The sequences were compared to the NCBI database by basic local alignment search tool and determined to be *F. heteroclitus* SGK1.

Dissociation curves were used to verify amplification of a single product. Initial amplicons were run on agarose gels to confirm product size and sequenced to ensure product specificity. Transcript abundance was calculated as copy number, based on serial dilution of a plasmid standard curve containing the entire killifish SGK1 sequence (pcDNA3.1-kf SGK1). Standard curves were linear over a 6-log range, and had a correlation coefficient close to 1 (R^2^≥0.99).

### Gill plasma membrane preparations

To measure gill plasma membrane CFTR, preliminary studies were conducted to modify a protocol to isolate plasma membranes [26]. Briefly, gill plasma membranes were isolated by cell lysis, removal of cellular debris followed by high-speed centrifugation. Freshwater acclimated adult killifish were injected with 14 µg/g translational blocking SGK1 vivo-morpholino, or control vivo-morpholino. Fish were returned to freshwater for 4 h with temperature control and aeration. After 4 h in freshwater, fish were transferred to 100% seawater for 1 h, except for freshwater control vivo-morpholino treated fish, which remained in freshwater. Fish were sacrificed by cervical dislocation, gills were surgically removed and gill filaments removed from bony arches. Gill plasma membranes were prepared based on a protocol from Flik et al. with minor modifications [26]. Gill tissue was homogenized in a hypotonic buffer (pH 8, 25 mM NaCl, 1 mM Hepes, 1 mM Tris- Base, 1 mM DTT, with EDTA free Complete protease inhibitor). Gills were first disrupted with a Tissue-Tearor, followed by thorough homogenization with a Dounce homogenizer and finally passed through a 22 G needle 10–15 times. Samples were centrifuged at 600×g for 15 minutes at 4°C to remove nuclei and cellular debris. After this centrifugation step, an aliquot of each sample was taken as whole cell lysate for analysis. The remaining supernatant was transferred to an ultra centrifuge tube and brought up to a volume of 4 ml with hypotonic homogenization buffer. Samples were spun at 175,000×g for 45 minutes at 4°C. The supernatant was removed and the entire pellet re-suspend in 1 ml of isotonic buffer (pH 7.4, 250 mM Sucrose, 5 mM MgCl_2_, 5 mM Hepes, 5 mM Tris-HCl, 1 mM DTT) with 100 strokes in a Dounce homogenizer with the loose pestle. The re-suspended pellet was differentially centrifuged at 1000×g for 10 minutes, then 10,000×g for 10 minutes and finally 50,000×g for 30 minutes, all at 4°C. Supernatant from the last centrifugation step was removed and the final pellet, containing the plasma membrane, was re-suspended with 50 µL of re-suspension buffer (pH 7.4, 20 mM Hepes, 20 mM Tris-HCl, 50 mM MgCl_2_, 150 mM KCl) by ten passages through a 22 G needle. All samples collected were combined with an equal volume of Laemelli buffer with 1 mM DTT and heat denatured at 85°C for 5 minutes prior to separating on 4–15% SDS-PAGE gel.

To assess the purity of the plasma membrane preparation Western blots studies were conducted using gill lysates from freshwater and seawater acclimated fish for Rab4a to examine endosomal contamination. A known transmembrane protein found in the killifish gill, Na^+^, K^+^-ATPase was examined by Western blot to determine enrichment of the membrane fraction [6].

### Statistics

Data presented are mean±SEM. Statistical significance was determined by one-way ANOVA followed by a Tukey's HSD post-hoc test using a p value<0.05 as significant Statistical analysis was performed with Prism v 5.0 (Graph Pad Software, San Diego, CA).
